# A Systematic Review of Patient Race, Ethnicity, Socioeconomic Status, and Educational Attainment in Prostate Cancer Treatment Randomised Trials—Is the Evidence Base Applicable to the General Patient Population?

**DOI:** 10.1016/j.euros.2023.05.015

**Published:** 2023-06-18

**Authors:** Siddhant Patki, Julian Aquilina, Rebecca Thorne, Isaac Aristidou, Filipe Brogueira Rodrigues, Hannah Warren, Axel Bex, Veeru Kasivisvanathan, Caroline Moore, Kurinchi Gurusamy, Mark Emberton, Lawrence M.J. Best, Maxine G.B. Tran

**Affiliations:** a Imperial College Medical School, London, UK; b University College London Medical School, London, UK; c The Leeds Teaching Hospitals NHS Trust, Leeds, UK; d North Middlesex University Hospital NHS Trust, London, UK; e University College London Division of Surgery and Interventional Science, London, UK; f Royal Free London NHS Foundation Trust, London, UK; g Northern Care Alliance NHS Trust, Salford, UK

**Keywords:** Prostate, Neoplasm, Disparity, Race, Socioeconomic, Education, Systematic, Randomised controlled trial

## Abstract

**Context:**

Prostate cancer (PC) disproportionately affects men of Black race, and lower educational and socioeconomic status. Guidelines are based on randomised controlled trials (RCTs); however, the representation of different races, educations, and socioeconomic backgrounds in these trials is unclear.

**Objective:**

To assess reporting of equality, diversity, and inclusion characteristics (Equality, Diversity and Inclusion [EDI]) and differences in treatment effects between different races, and educational or socioeconomic status.

**Evidence acquisition:**

We conducted a systematic review of CENTRAL, MEDLINE, and Embase in April 2020 examining RCTs investigating treatments for PC. Outcomes collected were race/ethnicity, educational attainment, and socioeconomic status. RCTs investigating PC treatment in any population or setting were included. Data extraction of characteristics was performed independently by pairs of reviewers and checked by a senior author. The Cochrane risk of bias tool assessed the quality of included papers.

**Evidence synthesis:**

A total of 265 trials were included, and 138 of these were available as full-text articles. Fifty-four trials including 19 039 participants reported any EDI data. All 54 trials reported race, 11 reported ethnicity, three reported educational attainment, and one reported socioeconomic status. Patients of White race were the majority of the recruited population (82.6%), while the minority prevalence was as follows: Black 9.8% and Asian 5.7%. Three studies reported mortality outcomes depending on the participant’s race. All three studies investigated different treatments, so a meta-analysis was not performed. No studies reported outcomes stratified by the educational or socioeconomic status of participants.

**Conclusions:**

There is poor reporting of patient race, ethnicity, socioeconomic background, and educational attainment in RCTs for PC treatments between 2010 and 2020. Addressing this for future studies will help explain differences in the incidence of and mortality from PC and improve the generalisability of results.

**Patient summary:**

In this study, we reviewed prostate cancer treatment trials to see whether these reported race, education, and socioeconomic backgrounds of their patient populations. We conclude that reporting of these characteristics is poor. This needs to be improved in future to improve outcomes for patients with prostate cancer of all ethnical, racial, and socioeconomic groups.

## Take Home Message

There is poor reporting of patient race, ethnicity, socioeconomic background, and educational attainment in randomised controlled trials for prostate cancer treatments between 2010 and 2020. Future studies need to collect these important demographic data to help explain differences in incidence and mortality from prostate cancer and improve the generalisability of results.

## 1 Introduction

Prostate cancer is the most frequently diagnosed cancer in men in the developed world, with >1.4 million cases and 375 000 deaths globally in 2020 [1]. Population-level data have shown the lifetime risk of developing prostate cancer to be one in eight for White men, one in four for Black men, and one in 13 for men of Asian ethnicity, while the risk of death from prostate cancer is one in 24 for White men, one in 12 for Black men, and one in 44 for men of Asian ethnicity in the UK [2]. Disparities in prostate cancer incidence and outcomes have both a genetic [3] and a socioeconomic basis [4], [5], [6], [7], with a complex interaction between structural, social, and health factors [8].

Clinical trials form the foundation of evidence-based medicine, from which best practice is determined for managing patients. Systematic reviews of randomised clinical trials (RCTs) are considered to provide the highest level of evidence (level 1a [9]). However, for trial results to be applicable to a given patient, study populations should reflect and be representative of those we see in clinical practice. There are well-documented racial and socioeconomic disparities in clinical trial populations, which compromises the generalisability of trial results [10]. The reason for this remains unclear, as evidence suggests equal willingness of under-represented groups to participate in medical research [11], [12]. Prostate cancer is a particularly pertinent example as it disproportionately affects men of Black race, and lower socioeconomic status and educational attainment [2], [4], [5], [6], [7], [8], [13]. In the UK, the National Institute for Health and Care Research published “Equality, Diversity and Inclusion (EDI) requirements” in 2020, stating the expectation that every person eligible to take part in research should be offered the same opportunities, regardless of socioeconomic status, location, or protected characteristics [14]. Grant applicants are expected to explain how EDI will be addressed, delivered, and documented in their studies. In the USA, since 2001, the National Institutes of Health (NIH) mandated appropriate inclusion of minority groups in all publicly funded research, and race and ethnicity are routine reporting domains on the ClinicalTrials.gov database [15].

### 1.1 What has been done

A recently published review of all prostate cancer trials found that 69.2% of trials run in the USA reported racial characteristics of included patients and found that Black and Hispanic patients were significantly under-represented [16]. In 2020, a review of the ClinicalTrials.gov registry to assess the diversity of enrolment in prostate cancer clinical trials up to 2016 was completed. Recently, the IRONMAN registry has been established to improve the deficiencies in this area of the evidence base.

### 1.2 Aim

Our primary aim was to investigate whether prostate cancer RCT outcomes are associated with EDI characteristics. Secondary aims included quantification of the reporting of EDI in all RCTs of prostate cancer treatment. We used race, ethnicity, socioeconomic status, and educational attainment as indicators of EDI. In addition, we sought to assess differences in treatment outcomes by each of these variables.

## 2 Evidence acquisition

### 2.1 Protocol

This systematic review was conducted in line with the Preferred Reporting Items for Systematic Reviews and Meta-analyses (PRISMA) guidelines [17] and was registered with PROSPERO (CRD42020189042).

### 2.2 Search strategy

We developed a comprehensive search strategy around key themes of randomised controlled trials and prostate cancer (Supplementary material) and searched CENTRAL, MEDLINE, and Embase from 2010 to 2020 on April 24, 2020.

### 2.3 Eligibility and study selection

Randomised controlled trials investigating the treatment of prostate cancer in any population or setting were included. Studies investigating the treatment of localised and metastatic prostate cancer were included. Studies investigating isolated treatment to bone metastases, symptom management, or explicitly palliative treatment, that is, without any treatment targeted at the prostate were excluded. We did not specify outcome reporting as part of our inclusion criteria. No language restrictions were imposed.

Two authors completed study selection independently (S.P. and J.A., R.T., or I.A.) for each study, with disagreements reviewed by a senior author (L.B.). Papers were selected based on the title and abstract, with a further screening of included studies once full texts were retrieved.

### 2.4 Data extraction

Data on the characteristics of included studies were extracted independently by pairs of reviewers (S.P. and J.A., I.A., or R.T.) and checked by a senior author (L.B.).

Data extracted for included studies comprised year of publication, authors, trial name/ID, journal impact factor, country(ies) data collected in, funder, whether the study was reported in a conference abstract or a full-text publication, and whether the following EDI characteristics of study populations were reported: race, ethnicity, socioeconomic status, or educational attainment. If EDI characteristics of the study population were not reported, no further data extraction was performed.

For studies that reported EDI domains, further data were extracted, including patient characteristics (age, cancer stage, ethnicity, socioeconomic group, and educational attainment), study intervention, eligibility criteria, number of participants in each trial arm, study outcomes (mortality, disease-free survival, adverse events, and health-related quality of life) by ethnicity, socioeconomic group, and educational attainment. Race and ethnicity were considered distinct entities as per reporting of results on ClinicalTrials.gov. This meant that Hispanic or Latino was considered to be an ethnicity as many ClinicalTrials.gov registrations report this distinctly to race characteristics. The risk of bias was assessed using the Cochrane risk of bias tool (ROB1).

### 2.5 Planned analyses

The primary analysis was to assess the outcome associated with EDI data. A meta-analysis was planned to investigate the differences in outcomes for patients of different racial, ethnic, socioeconomic, and educational attainment backgrounds. Measures of effect were planned as described in the *Cochrane handbook for intervention reviews* [18]. All forest plots were generated using RevMan software [19].

We planned to assess several variables for their association with reporting of EDI characteristics: journal impact factor (2020 if available) and mean study size as potential surrogate markers of research quality, funding body (public vs commercial), and publication date to assess for changes in reporting over time. Two-tailed *T* tests were performed to compare means, Pearson correlation coefficient was used to assess changes over time, and a chi-square test was performed to compare the races of included trial participants with those affected in the general population.

## 3 Evidence synthesis

### 3.1 Search results

**Fig. 1:**
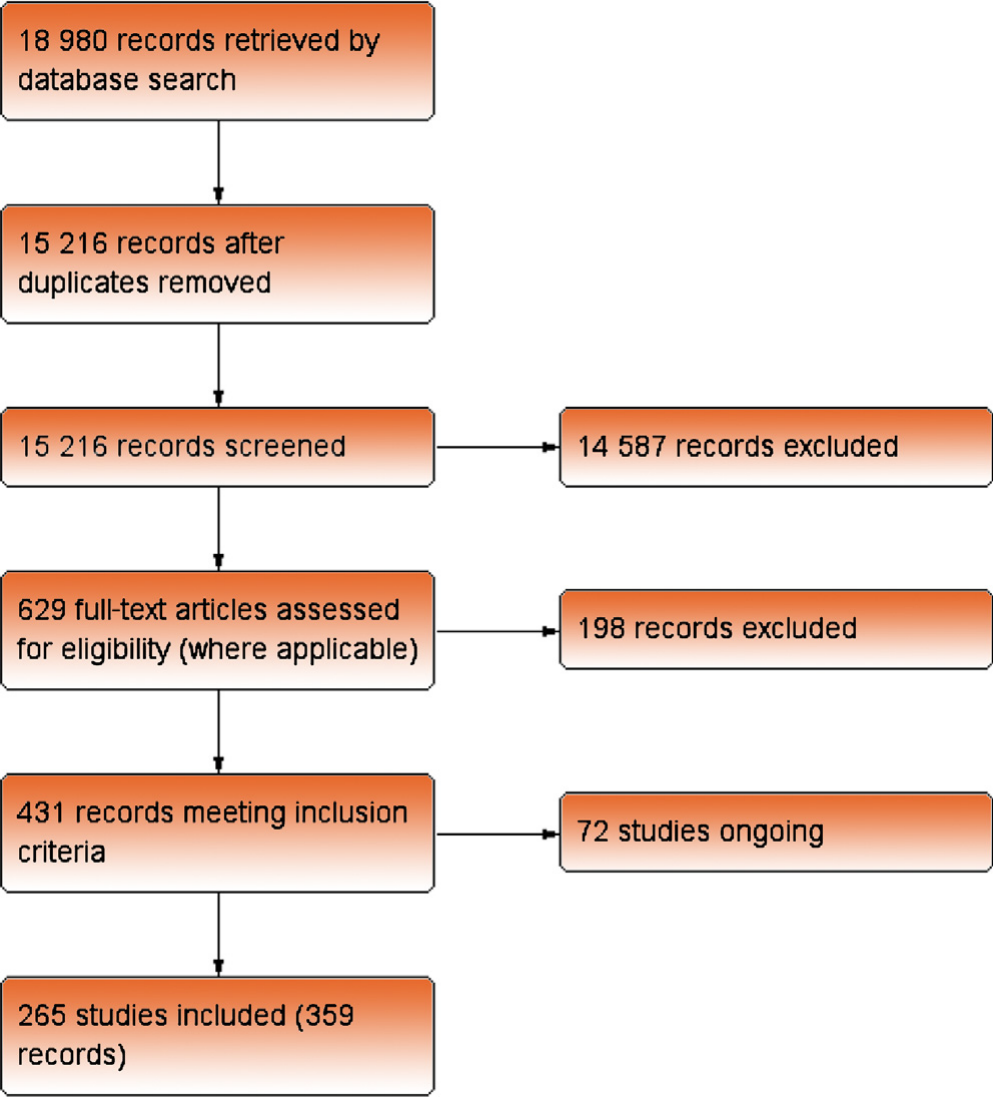
Flow diagram of the study selection process and reasons for exclusion from the meta-analysis.

The search returned 18 980 records, of which 3764 were duplicates. After title and abstract screening, 629 full-text articles were assessed for eligibility. From these, a further 198 records were excluded. This resulted in 437 references reporting 337 studies. A single study may have multiple references but would be included only once in the analysis to prevent inclusion of an individual trial participant multiple times. Seventy-two studies were on-going and therefore did not contribute data to this review, leaving 265 included studies. The study flow is summarised in Figure 1.

### 3.2 Summary of included study characteristics

**Fig. 2:**
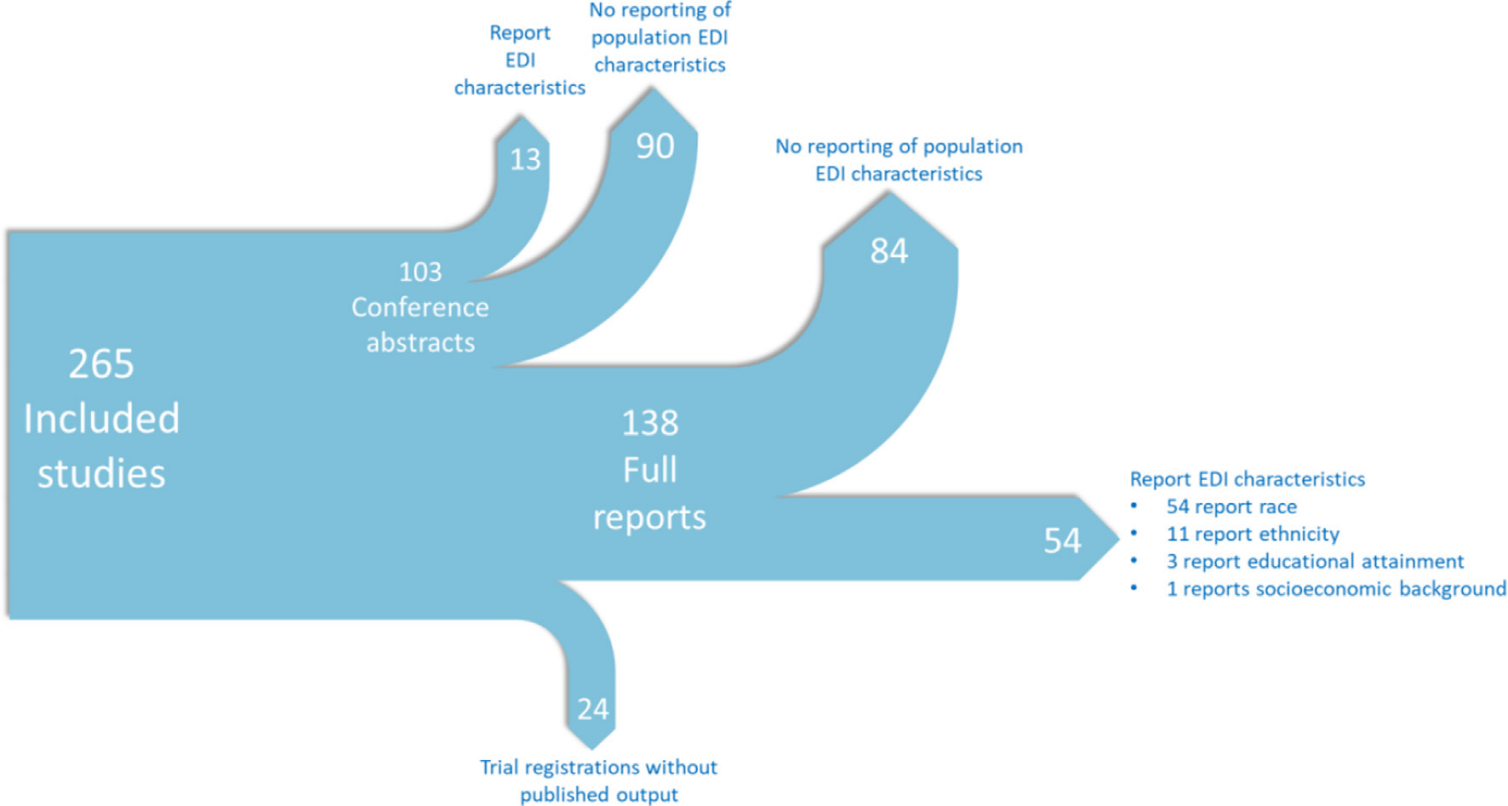
Summary of included study characteristics. EDI = Equality, Diversity and Inclusion.

The characteristics for included trials can be seen in Figure 2. Of the 265 studies included, 138 were full-text papers, 103 conference abstracts, and 24 trial references. Here, we focus on results from included studies that have a full-text report (“full-text studies”), while the results from all paper types including conference abstracts are presented in the Supplementary material. Of the 138 full-text studies including 49 068 participants, 54 including 19 039 participants (39% of both papers and participants) reported any EDI data. Of these, all 54 reported race and 11 reported ethnicity, with one paper reporting socioeconomic data and three papers reporting educational attainment of the participants. All studies reporting ethnicity, socioeconomic status, or educational attainment also reported race.

#### 3.2.1 Risk of bias

The 54 full-text studies reported that minorities were assessed for the risk of bias using the Cochrane risk of bias tool. Thirty-five of the 54 studies had at least one domain that was considered to be at a high risk of bias.

#### 3.2.2 Racial breakdown

**Fig. 3:**
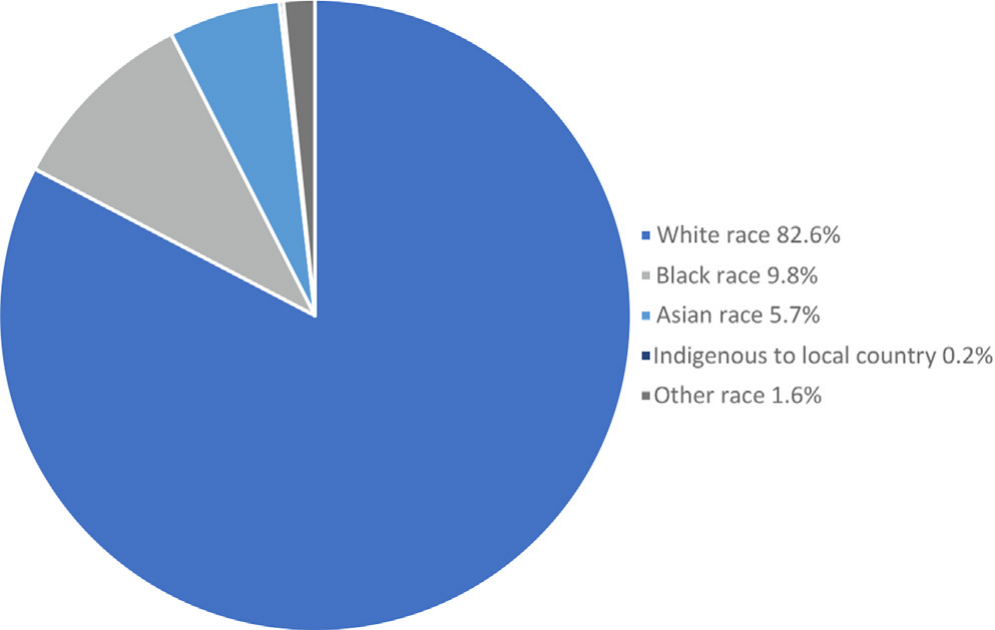
Summary of population demographics by race across the 54 studies reporting participant racial characteristics.

Of the 54 studies reporting EDI data, a summary of population demographics by race is shown in Figure 3. Overall, patients of White race make up the vast majority of patients recruited for prostate cancer trials at almost 82.6% across all studies. In comparison, Black patients made up 9.8% and Asian patients made up 5.7% of the combined study populations.

#### 3.2.3 Ethnicity breakdown

Hispanic ethnicity was reported by 11 papers. In those papers reporting ethnicity, 7.9% of patients were Hispanic, and 92.1% were non-Hispanic (711 Hispanic vs 8318 non-Hispanic participants).

#### 3.2.4 Mean study size

Mean study sizes were similar between included studies with a full text, which reported and did not report any EDI data (353 ± 63.2 participants vs 358 ± 57.3 participants, *p* = 0.955).

#### 3.2.5 Impact factor

Papers reporting EDI data were published in journals with a higher impact factor in comparison with papers that did not report EDI data; however, this was not statistically significant (27.4 ± 3.8 vs 19.7 ± 2.4, *p* = .0.069).

#### 3.2.6 Funding body

Commercial studies had a statistically significantly greater likelihood of reporting population EDI data than trials with noncommercial funders (59% ± 6.5% vs 35% ± 6.7%, *p* = 0.012).

#### 3.2.7 Number of trials by country

The geographic location of included studies is reported by the nation in which they were undertaken in Table 1. The largest number of trials in an individual country was conducted in the USA (36 trials). Forty-three multinational trials were included.

**Table 1:** Geographical location of included studies

	Number of included studies by country	Number of studies reporting EDI by country	Percentage
**Australia**	4	2	50
**Belgium**	1	0	0
**Canada**	8	0	0
**China**	2	0	0
**Finland**	2	0	0
**France**	4	0	0
**Germany**	3	0	0
**Greece**	1	0	0
**Ireland**	2	0	0
**Italy**	4	0	0
**Japan**	5	1	20
**Multinational**	43	26	60
**Netherlands**	6	0	0
**Not stated**	1	0	0
**South Korea**	2	0	0
**Spain**	2	0	0
**Sweden**	3	0	0
**Turkey**	1	0	0
**UK**	8	0	0
**USA**	36	25	69

EDI = Equality, Diversity and Inclusion.

#### 3.2.8 Likelihood of reporting population EDI data by country

Multinational studies are more likely to report race or ethnicity data than RCTs carried out in a single country (60.5% ± 7.5 vs 29.5% ± 4.7, *p* = 0.044). Few single-nation studies outside the USA reported race/ethnicity data (5.2% ± 2.9% [non-USA single-nation studies] vs 69.4% ± 7.8% [US studies], *p* = 0.000).

#### 3.2.9 Changes in reporting over time

**Fig. 4:**
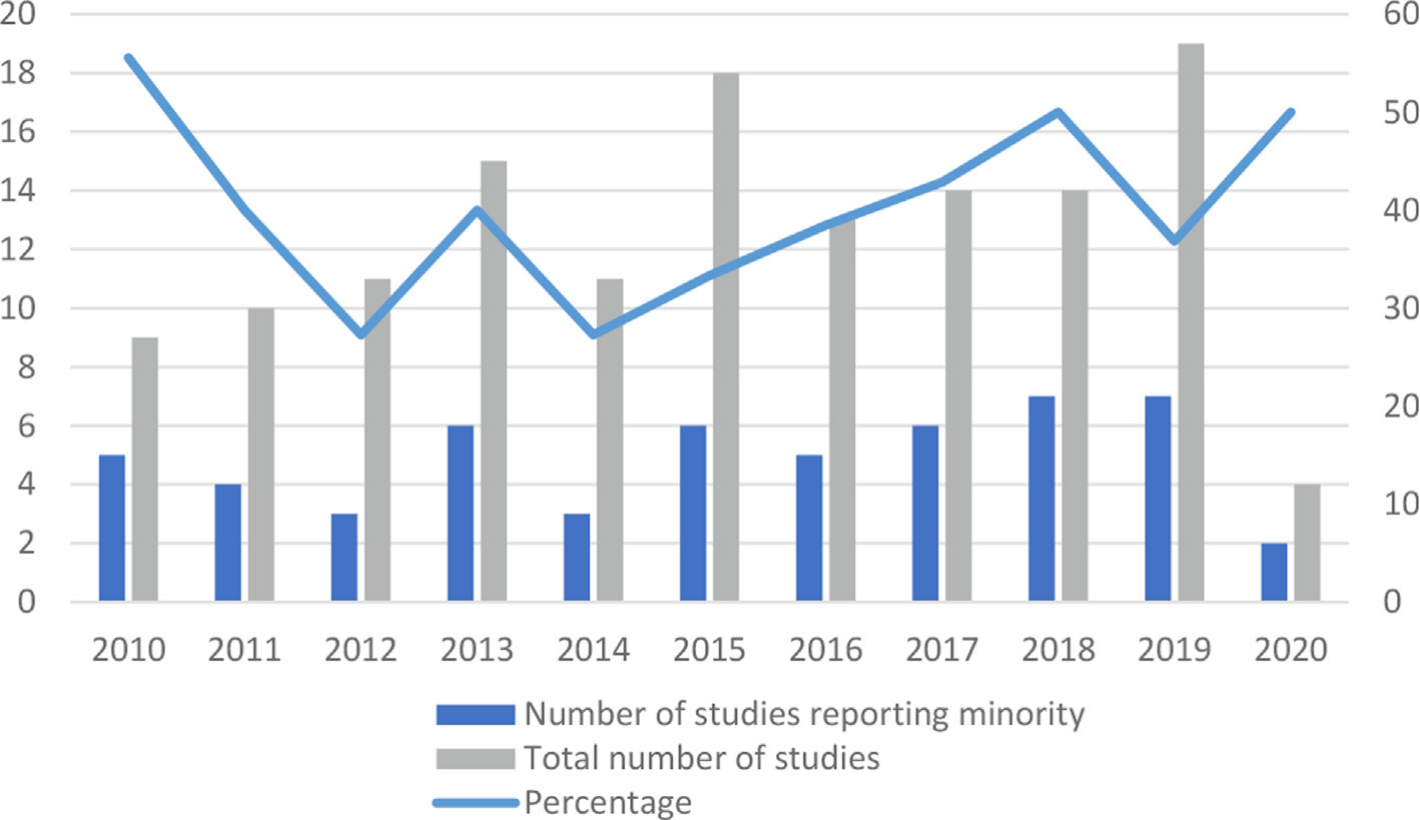
Temporal trend in reporting of EDI data of study populations. EDI = Equality, Diversity and Inclusion.

The proportion of studies that reported race or ethnicity data did not change over time (Pearson correlation –0·19, *p* = 0·958), as shown graphically in Figure 4. Note that the year given is of the primary reference for the publication of the study and not the year in which the study was conducted.

#### 3.2.10 Comparison of mortality by ethnic group

**Fig. 5:**
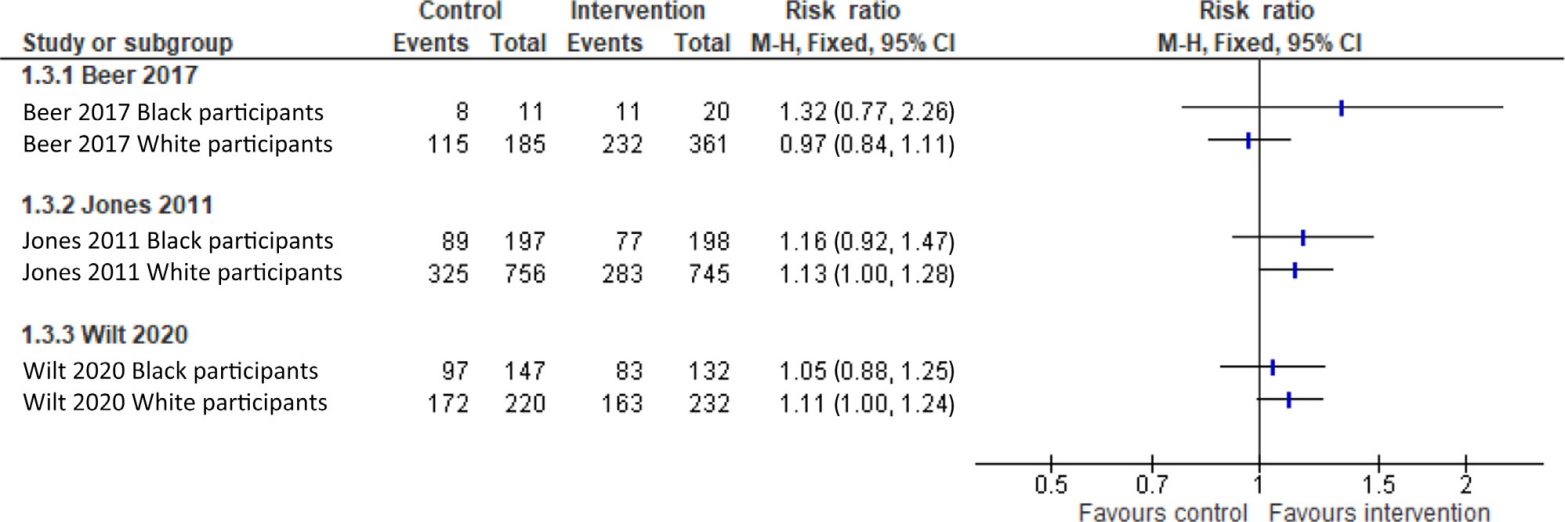
Forest plot of mortality outcome by race for participants in included RCTs. RCT = randomised clinical trial. CI = confidence interval; M-H = Mantel-Haenszel.

Only four studies reported outcomes stratified by any EDI [20], [21], [22], [23]. Three of these reported the binary mortality for participants of Black versus White race and have graphically been presented in Figure 5. These studies investigated three different treatments; therefore, a meta-analysis is inappropriate. One study reported mortality as a time-to-event outcome for participants of Black versus White race, which has not been presented. The forest plots show a good overlap of confidence intervals in the effect of the intervention between Black and White participants, indicating no evidence of difference in outcomes.

#### 3.2.11 Comparison of prostate cancer enrolment to patient population within the USA

A statistical comparison of the EDI of trial participants in each country with the incidence of prostate cancer by EDI in the general population of that country was planned. However, for trials performed in single countries, the USA was the only country with more than two RCTs that reported EDI data. The multinational studies did not report recruitment in enough detail to allow for the proportion of participants from each country to be calculated.

A chi-square test was used to compare the proportions of White, Black, and Asian participants recruited by prostate cancer trials within the USA with the incidence of prostate cancer in people of White, Black, and Asian race within the general population of the USA. Data were retrieved from the Centers for Disease Control and Prevention Morbidity and Mortality Weekly Report of October 16, 2020, and the result is presented in Table 2. There was a statistically significant difference in the proportion of different races between the trials and US cancer incidence (chi-square = 32.626, *p* < 0.0001). From the table of percentages, the main difference was due to under-representation of Asian races in clinical trials.

**Table 2:** Comparison of the number of participants of White and Black race in prostate cancer trials conducted within the USA with the number of people of White and Black race affected by prostate cancer from 2001 to 2017, as reported by the CDC

	White race	Black race	Asian race
*Population included in trials of prostate cancer treatment*	2970 (82.5%)	586 (16.3%)	29 (0.8%)
*Total population affected in the USA in 2001–2017*	2 296 805 (81.4%)	451 822 (16.0%)	62 184 (2.2%)

CDC = Centers for Disease Control and Prevention.

### 3.3 Assessment of EDI reporting in NICE guidelines

National Institute for Health and Care Excellence (NICE) guideline 131 (NG131) describes recommendations for prostate cancer diagnosis and management. We investigated the reporting of EDI within the trials used to make decisions regarding prostate cancer treatment guidelines. Within NG131, there are three relevant treatment comparisons. Overall, six out of 17 trials (35·2%), which were used for the three treatment recommendations, reported EDI. These studies that reported the race of included participants included 84% participants of White race, 12.3% of Black race, 0.7% of Asian race, and 3% of “other” racial category.

### 3.4 Reporting bias

An assessment of the reporting bias by a funnel plot was not possible because of insufficient numbers of studies reporting relevant outcomes. Of the 265 studies included, 24 were trial registrations without any published output at all and a further 103 had only a conference abstract as the published output. However, we do not expect that the decision not to publish at all or publish only as a conference abstract was related to the differences (or lack of differences) in treatment effects related to the EDI characteristics.

## 4 Conclusions

This systematic review has demonstrated poor reporting of participant demographics in terms of race, ethnicity, socioeconomic group, and educational attainment in RCTs of prostate cancer treatment published between 2010 and 2020. Of all trials of prostate cancer treatment published in this time frame, only 39%, which were published as a full text, reported an EDI domain, and the vast majority of these reported only race. Socioeconomic demographics and educational attainment of participants are rarely reported.

Inadequate reporting limits the ability to identify and improve upon disparities in the population demographics of prostate cancer clinical trials. It also undermines our ability as clinicians to determine the generalisability of trial results to the patients who we see in clinical practice. Furthermore, failure to report treatment effect by EDI domains at study level precludes the use of statistical methods that could provide valuable evidence of best practice in specific populations in whom disparities in prostate cancer outcomes are known to exist. It is unclear why such differences in incidence and mortality from prostate cancer in different socioeconomic groups occur. Two possible broad reasons for this could be that first, there is reduced engagement with or access to healthcare by certain groups, and second, there are underlying disease- or socioeconomic status–driven differences between groups, which means that even with the exact same care, some EDI groups will do worse. Improvement in reporting of EDI domains would allow for the testing and delineation of these hypotheses for why, for example, patients of Black race have higher prevalence and mortality from prostate cancer. Addressing this research gap should be an urgent priority.

The minority of studies that reported the proportion of participants from different racial and ethnic groups must be interpreted in the context of the demographic of local populations affected by prostate cancer. In the UK, men of Black race made up 3.4% and Asian men 1.9% of prostate cancer diagnoses (data from Public Health England). In the USA, 16% of prostate cancer diagnoses are in Black men and 2% in Asian men [24]. A comparison of included participants in trials performed in the USA indicated that these were representative of the local population in terms of the proportion of people of White and Black race affected by prostate cancer in the American population. However, this is very different from the percentages in other countries.

NICE guidelines draw upon data from various countries. Only 35% of trials used to recommend prostate cancer treatments reported any EDI data. Of those that reported race, very few Asian participants were included, representing 0.7% of participants included in those studies that reported race.

There was no statistically significant evidence of difference in the impact factor of papers reporting EDI data versus those that did not. This may reflect a lack of power to demonstrate a difference, but also highlights on-going uncertainty regarding journal impact factor and research quality.

The principal limitation of this review is that authors of studies that report EDI data may be biased towards those with more inclusive recruitment. Therefore, the actual percentage of participants of various races included in prostate cancer RCTs may be significantly different from that reported here. In addition, it is not possible to comment on the socioeconomic background of trial participants because of the near universal lack of reporting of data on this. In our exploratory analysis summarised in Table 2, the exact time periods did not overlap and the trial participants were likely part of the total number; therefore, the results should be considered indicative rather than definitive.

Our priority was to understand the landscape of EDI reporting to allow a relevant standard against which to measure the effect of future initiatives, and therefore our review was limited to the trials published between 2010 and 2020.

Commercial studies were associated with improved EDI reporting. This may be the results of better resourcing or routine registration on trials databases that require racial demographic data. Promotion of registration of all trials on databases may therefore improve reporting of EDI characteristics.

Multinational trials were more likely to report study population race data in comparison with trials conducted in a single country. Out of all single-nation studies, those conducted in the USA most frequently reported population race and ethnicity—in 25/36 studies (69.4%). Factors contributing to improved reporting in the USA may include the NIH mandate for appropriate inclusion of minorities in publicly funded research, and that on reporting results of trials registered with the national trials registry ClinicalTrials.gov, population race and ethnicity are requested as standard.

A previous review by Rencsok et al [25] reported that racial characteristics of study populations were available for 81.9% of the 72 prostate cancer trials registered with ClinicalTrials.gov between 1987 and 2016, compared with 39% in the current review. This difference is in part explained by Rencsok et al [25] approaching investigators of included studies for race and ethnicity data where these were not publicly available, although the proportion of studies for which data were collected in this way was not reported. Additionally, identification of trials exclusively through the US National Library of Medicine’s ClinicalTrials.gov will generate a USA-centric dataset. Rencsok et al’s [25] review included only four studies (6%) that did not recruit participants from the USA, compared with 61/138 (44%) in the current review. We found higher rates of reporting of racial characteristics in exclusively US-based studies (25/36, 69·4%) compared with other single-nation studies (3/58, 5.2%), suggesting that the difference in geographic distribution of included studies may largely be responsible for the difference. In addition to providing a global rather than USA-centric perspective, a key strength of the current review is that it comprehensively included RCTs on prostate cancer treatment.

Eight studies conducted in Asian-majority nations [26], [27], [28], [29], [30], [31], [32], [33] that did not report participant race or ethnicity might be assumed to include a majority of Asian participants, allowing cautious generalisation of results from these trials to populations in these nations. However, the absence of clinical trials conducted in Black-majority nations severely limits the evidence base for Black men worldwide. This finding corresponds with the recommendations in the literature stating that incorporation of these countries is of vital importance to increase the representation of Black ethnic minorities in prostate cancer trials [24].

Since 1990, the percentage of White participants in prostate cancer study cohorts has not dropped below 80% [25]. This is reinforced by the lack of a statistically significant trend in the likelihood of reporting EDI indicators over time found in this review. The current review is the largest of its type (265 included studies), including all RCTs over the most recent decade. With the field of prostate cancer research developing rapidly, it provides a basis for the need for diversity and inclusion in studies for the future. The COVID-19 pandemic will have caused delays in diagnosis and treatment across the board, which may be dependent on EDI characteristics, further exacerbating this issue.

### 4.1 Research implications

We strongly advocate reporting of race/ethnicity, socioeconomic status, and educational attainment in prostate cancer RCTs. We would advocate the inclusion of a table with these characteristics in publications from an RCT, which could be included as a supplementary appendix. As a minimum, an indication of whether these data were collected or not should be specified.

We would recommend that changes be made in reporting standard guidelines for RCTs such as the CONSORT guidelines to reflect this. Further, consensus should be derived on accepted measurement tools for these EDI characteristics so that a comparison between studies is possible and investigators can more easily include appropriate data points or questionnaires in their studies.

### 4.2 Policy implications

Policies to ensure that RCTs recruit a relevant but diverse population are strongly recommended. This can be achieved through mandatory public funding body requirements for EDI characteristic inclusion at the grant funding application stage and mandatory reporting requirements by these grant bodies.

### 4.3 Future implications

This is the first systematic review and meta-analysis of RCTs on prostate cancer treatment specifically investigating reporting of EDI characteristics of race, ethnicity, socioeconomic status, and educational attainment and their association with clinical outcomes. The findings demonstrate poor reporting of EDI demographics in prostate cancer RCTs during the 10-yr period studied, underscoring the limitations of the generalisability of the evidence base driving current prostate cancer treatment decisions made by clinicians and health service providers.

### 4.4 Quality of evidence

Of the 54 studies reported as full text, which reported EDI criteria, 35 had at least one domain that was at a high risk of bias. Therefore, even in the papers that reported EDI criteria, 65% were considered to be at a high risk of bias.

  ***Author contributions*:** Maxine G.B. Tran had full access to all the data in the study and takes responsibility for the integrity of the data and the accuracy of the data analysis.

  *Study concept and design*: Tran, Rodrigues, Best.

*Acquisition of data*: Patki, Aquilina, Thorne, Aristidou.

*Analysis and interpretation of data*: Tran, Best.

*Drafting of the manuscript*: Tran, Best, Patki, Warren.

*Critical revision of the manuscript for important intellectual content*: Tran, Best, Emberton, Gurusamy, Moore, Kasivisvanathan, Bex.

*Statistical analysis*: Best, Gurusamy.

*Obtaining funding*: None.

*Administrative, technical, or material support*: None.

*Supervision*: Tran.

*Other*: None.

  ***Financial disclosures:*** Maxine G.B. Tran certifies that all conflicts of interest, including specific financial interests and relationships and affiliations relevant to the subject matter or materials discussed in the manuscript (eg, employment/affiliation, grants or funding, consultancies, honoraria, stock ownership or options, expert testimony, royalties, or patents filed, received, or pending), are the following: Kurinchi Gurusamy is a member of Equality, Diversity, and Inclusion committee at UCL Faculty of Medical Sciences and Deputy Lead for Athena Swan Committee at the Division of Surgery and Interventional Science. His promotions and salary depend upon conducting and reporting high-quality and high-impact articles. No other authors recorded competing interests.

  ***Funding/Support and role of the sponsor*:** None.

  ***Data sharing*:** No additional data. Extraction forms are available at request.
