# Supplementary material for: A Systematic Review of Patient Race, Ethnicity, Socioeconomic Status, and Educational Attainment in Prostate Cancer Treatment Randomised Trials—Is the Evidence Base Applicable to the General Patient Population?
Source: Eur Urol Open Sci. 2023 Jun 18;54:56–64. doi: 10.1016/j.euros.2023.05.015 (PMC10403690; doi:10.1016/j.euros.2023.05.015)
Supplement: Supplementary data 1 [file mmc1.docx]

# **Supplementary material 1**

# **Search strategy**

Date of searches: 24 April 2020

CENTRAL (via the Wiley platform):

1. MeSH descriptor: [Prostatic Neoplasms] explode all trees
2. (prostat* NEAR/2 (cancer OR neoplas*)):ti,ab
3. #1 OR #2 with Publication Year from 2010 to 2020, in Trials

MEDLINE (via Ovid SP):

- 1. exp Prostatic Neoplasms/
  2. (prostat* adj2 (cancer or neoplas*)).ab,ti.
  3. or/1-2
  4. randomized controlled trial.pt.
  5. controlled clinical trial.pt.
  6. randomized.ab.
  7. placebo.ab.
  8. clinical trials as topic.sh.
  9. randomly.ab.
  10. trial.ti.
  11. or/4-10
  12. exp animals/ not humans.sh.
  13. 11 not 12
  14. and/3,13
  15. limit 14 to yr="2010 - 2020"

Embase (via Ovid SP):

1. exp Prostatic Neoplasms/
2. (prostat* adj2 (cancer or neoplas*)).ab,ti.
3. or/1-2
4. (random$ or placebo$ or single blind$ or double blind$ or triple blind$).ti,ab.
5. RETRACTED ARTICLE/
6. or/4-5
7. (animal$ not human$).sh,hw.
8. (book or conference paper or editorial or letter or review).pt. not exp randomized controlled trial/
9. (random sampl$ or random digit$ or random effect$ or random survey or random regression).ti,ab. not exp randomized controlled trial/
10. or/7-9
11. 6 not 10
12. and/3,11
13. limit 12 to yr="2010 - 2020"

# **R code for chi-squared test**

df <- data.frame(White = c(2970,2296805),Black = c(586,451822),Asian = c(29,62184),row.names = c('USA-trials','USA-prostate cancer new cases'))

chisq.test(df)
