# Supplementary material for: A Systematic Review of Patient Race, Ethnicity, Socioeconomic Status, and Educational Attainment in Prostate Cancer Treatment Randomised Trials—Is the Evidence Base Applicable to the General Patient Population?
Source: Eur Urol Open Sci. 2023 Jun 18;54:56–64. doi: 10.1016/j.euros.2023.05.015 (PMC10403690; doi:10.1016/j.euros.2023.05.015)
Supplement: Supplementary data 2 [file mmc2.docx]

**Supplementary material 2: Details from all included studies (including conference abstracts)**

Summary of included studies (non-full text i.e. conference abstracts)

From the 265 included studies, 103 were conference abstracts and 24 were trial registrations without published output. From the 74 included studies that reported EDI data, 54 were full text studies, 13 were conference abstracts and 7 were trial registrations.

Of those reporting minorities (not full text):

From the 20 conference abstracts and trial registrations, including a total of 10814 randomised patients, 20 reported race, 13 reported ethnicity, 0 reported socio-economic data, 0 reported educational attainment.

Risk of Bias

Of the 20 included studies that were not full text (i.e. conference abstracts or NCTs/references/reports), 14 had at least one domain which was considered to be at high risk of bias. This takes into account the large proportion of assessments that were unclear due to the lack of information.

Racial Breakdown

For any paper type (i.e. all 74 included studies reporting EDI data), patients of white race continued to make up the majority of patients recruited for prostate cancer trials at 82.7%. The majority of the rest of the patient population were black and Asian patients making up around 8.5% and 6.5% respectively.

Mean Study Size

Mean study sizes varied between included studies of any paper type which reported and did not report any EDI data (did report = 375.7 ± 57.1; did not report = 304.1 ± 33.2 {p = 0.257})

Impact Factor

The trend seen with full-text studies is also seen with any paper type as papers reporting EDI data were published in journals with a higher impact factor in comparison to papers that did not report EDI data (did report = 31.1 ± 3.2; did not report = 23.0 ± 1.6 {p = 0.013})

Funding Body

For any study type, privately funded studies had a greater likelihood of reporting population EDI data than publicly funded (or no funding) trials (51% ± 5.5; 29% ± 4.8 p = 0.002)

Number of trials by country

|  | Number of included studies by country | Number of studies reporting any EDI characteristic by country |
| --- | --- | --- |
| Australia | 5 | 2 |
| Austria | 1 | 0 |
| Belgium | 2 | 0 |
| Canada | 13 | 0 |
| China | 9 | 0 |
| Finland | 3 | 0 |
| France | 11 | 1 |
| Germany | 7 | 0 |
| Greece | 1 | 0 |
| India | 2 | 0 |
| Ireland | 2 | 0 |
| Italy | 9 | 0 |
| Japan | 10 | 1 |
| Kazakhstan | 1 | 0 |
| Multinational | 56 | 25 |
| Netherlands | 8 | 0 |
| Norway | 1 | 0 |
| Not stated | 16 | 0 |
| Russia | 1 | 0 |
| South Korea | 3 | 0 |
| Spain | 4 | 0 |
| Sweden | 4 | 0 |
| Taiwan | 1 | 0 |
| Turkey | 1 | 0 |
| UK | 12 | 1 |
| USA | 82 | 44 |

Likelihood of reporting population EDI data by country

For all study types, multinational studies were more likely to report population EDI data than RCTs carried out in a single country (44.6% ± 6.7 vs 23.4% ± 2.9 p = 0.002)

Table with characteristics of all included trials which reported EDI data (with and without full text)

In total 74 studies reported EDI data – 54 full text studies, 13 conference abstract studies and 7 studies with just a NCT registration.

Abbreviations

- mcrpc = metastatic castrate resistant prostate cancer
- nct = national clinical trial
- TASQ = tasquinimod
- NHT = neoadjuvant hormonal therapy
- EDI = equality, diversity, inclusion?

| **Study Name (first author and year)** | **Journal Impact Factor** | **Country** | **Trial ID** | **Conference Abstract?** | **Prostate Cancer Stage** | **Total randomised patients** | **Intervention 1** | **Intervention 2** | **Intervention 3** | **Intervention 4** | **Ethnicity split** | **Socio economic group split** | **Education split** |
| --- | --- | --- | --- | --- | --- | --- | --- | --- | --- | --- | --- | --- | --- |
| van Die et al., 2017^1^ | 4.10 | Australia | ANZCTR number: 366895 | no | biochemically recurrent PC | 20 | placebo | active |  |  | 18 Caucasian, 2 Asian |  | (8) year 11 or under education, (5) year 12, (6) TAFE or university, (1) postgraduate |
| Yaxley et al., 2016^2^ | 79.3 | Australia | ACTRN12611000661976 | no | localised | 326 | radical retropubic prostatectomy | robot-assisted laparoscopic prostatectomy |  |  | 193 UK/Irish, 31 European, 3 indigenous Australian, 8 Asian, 4 pacific islander, 17 other | household income more than $60,000 (88), household income less than $60,000 (159) | (30) college educate; (229) high school educate or lower |
| Oudard 2017^3^ | 7.45 | France | NCT01308567 | no | mcrpc | 1168 | receiving C20 | receiving C25 | receiving D75 |  | 1088 white, 23 black, 47 Asian and 10 other |  |  |
| Matsubara et al., 2019^4^ | 3.40 | Japan | NCT02043678 | no | mcrpc | 114 | AAP + radium-223 | AAP + placebo |  |  | 113 Asian 1 American Indian or Alaskan native |  |  |
| Beer et al., 2017^5^ | 44.5 | Europe, USA, South America, and Australia. | NCT01057810 | no | mcrpc | 602 | Ipilimumab | Placebo |  |  | 546 white, 31 black, 6 Asian, 3 American Indian/Alaskan, 1 native Hawaiian, 15 other |  |  |
| De Bono et al., 2010^6^ | 79.3 | Multinational - East and West | NCT00417079 | no | mcrpc | 755 | mitoxantrone | Cabazitaxel |  |  | 631 white, 58 Asian, 40 black, 26 other |  |  |
| De Bono et al., 2014^7^ | 12.5 | Multinational - West | NCT00313781 | no | mcrpc | 204 | Figitumumab þ docetaxel/ prednisone | Docetaxel/ prednisone alone | Figitumumab þ docetaxel/prednisone (crossover from B1 |  | 226 white, 7 black, 8 other |  |  |
| Fizazi et al., 2017b^8^ | 33.0 | Multinational - Europe | NCT01732549 | no | M1 | 144 | Tasquinimod | Placebo |  |  | 110 Caucasian, 1 black/African American, 1 multiple races, 32 missing |  |  |
| Hakenberg et al., 2019^9^ | 9.16 | Europe | NCT01204710 | no | mcrpc | 123 | Olaratumab + M/P | M/P |  |  | 120 white, 1 more than one race |  |  |
| Heidenreich et al., 2013^10^ | 33.0 | Multinational |  | no | mcrpc | 131 | Docetaxel + prednisone + placebo | Docetaxel + prednisone + intetumumab |  |  | 107 white, 21 Asian, 3 black |  |  |
| Pili et al., 2011^11^ | 44.5 | USA, Canada and Sweden |  | no | mcrpc | 206 | tasquinimod | placebo |  |  | 173 white, 2 Asian, 22 black/African American, 4 other |  |  |
| Shore et al., 2016^12^ | 79.3 | USA, Belgium, Canada, Denmark, France, Germany, Romania, UK | NCT01288911 TERRAIN, | no | mcrpc | 375 | enzalutamide | bicalutamide |  |  | 348 white, 18 black or African American, 5 Asian, 2 native Hawaiian or other pacific islander, 2 other |  |  |
| Smith et al., 2016^13^ | 44.5 | USA, Australia, Austria, Belgium. Canada, France, Germany, Ireland, Italy, Netherlands, Puerto Rico, Spain, Sweden, UK | NCT01605227 | no | mcrpc | 1028 | Cabozantinib | Prednisolone |  |  | 785 white, 20 black or African American, 2 Asian, 1 American Indian or Alaskan native, 9 other, 211 not reported |  |  |
| Sonpavde et al., 2012^14^ | 33.0 | USA and Russia | NCT00286793 | no | mcrpc | 221 | docetaxel plus prednisone | docetaxel plus prednisone plus AT-101 |  |  | 205 white. 9 African American, 6 not reported |  |  |
| Tannock et al., 2013c^15^ | 79.3 | Multinational - East and West | NCT00519285 VENICE, | no | mcrpc | 1224 | Aflibercept | Placebo |  |  | 1112 Caucasian/white, 32 black, 68 Asian, 12 other |  |  |
| Wiechno et al., 2014^16^ | 18.7 | USA, Germany, Poland, Spain | NCT00642018 | no | mcrpc | 154 | LY2181308 sodium/Docetaxel | Docetaxel alone |  |  | 143 Caucasian, 4 African, 1 Hispanic, 1 east Asian |  |  |
| Ye et al., 2017^17^ | 2.78 | China. Malaysia, Russia, Thailand | NCT01591122 | no | mcrpc | 313 | Abiraterone + prednisolone | Placebo + prednisolone |  |  | 244 Asian, 69 white |  |  |
| Yu et al., 2015^18^ | 44.5 | USA and Canada | NCT01120236 | no | New hormone sensitive metastatic prostate cancer | 210 | Androgen Deprivation Combined with Cixutumumab | Androgen Deprivation Alone |  |  | 182 white, 14 black, 14 other |  |  |
| Yu et al., 2018^19^ | 3.85 | USA and Canada | NCT01120470 | no | mcrpc | 74 | prednisolone alone | apatorsen plus prednisolone |  |  | 69 white |  |  |
| Hussain et al., 2018^20^ | 91.2 | Multinational - East and West | NCT02003924 PROSPER | no | Adenocarcinoma of the prostate | 1401 | Enzalutamide 160 mg | Placebo |  |  | 991 white, 230 Asian, 31 black, 5 native Hawaiian, 8 multiple, 20 other |  |  |
| Roach et al., 2018^21^ | 79.3 | USA and Canada | NCT00769548 | no | localized (Any stage with risk of node involvement >15% ) | 1322 | Neoadjuvant total androgen suppression (TAS) (NHT) given 2 months before and during radiation therapy (RT) to the whole pelvis followed by a prostate boost. Drug: flutamide Drug: goserelin acetate Radiation: low-LET photon therapy | Neoadjuvant TAS (NHT) given 2 months before and during RT to the prostate only. Drug: flutamide Drug: goserelin acetate Radiation: low-LET photon therapy | RT to the whole pelvis followed by a boost to the prostate followed by 4 months of TAS (AHT) Drug: flutamide Drug: goserelin acetate Radiation: low-LET photon therapy | RT to the prostate only followed by 4 months of TAS (AHT) Drug: flutamide Drug: goserelin acetate Radiation: low-LET photon therapy | 881 white, 37 Hispanic or Latino, 322 black or African American |  |  |
| Sternberg 2016^22^ | 44.5 | Multinational - East and West | NCT01234311 | no | mcrpc | 1245 | Tasquinimod | placebo |  |  | 729 white 20 black 46 Asian 37 other Hispanic vs non-Hispanic also reported |  |  |
| Clarke et al., 2018^23^ | 41.3 | UK | NCT01972217 | no | mcrpc | 142 | olaparib plus abiraterone | placebo plus abiraterone |  |  | 134 white, 2 black or African American, 1 Asian, 5 other |  |  |
| Antonarakis et al., 2017a^24^ | 44.5 | USA | NCT01718353 | no | M1 | 63 | Docetaxel | Cabazitaxel |  |  | 55 Caucasian/white, 7 black, 1 Asian |  |  |
| Beard et al., 2011^25^ | 6.07 | USA |  | no | non metastatic prostate cancer | 54 | Reiki | RRT | Control |  | 49 white |  | (40) college educate; (14) high school educated or lower |
| Beer et al., 2013b^26^ | 2.65 | USA | NCT00779402 | no | T1b - T3c, N0 - N1, Nx, or M0 | 176 | sipuleucel-T | control |  |  | 159 white, 12 African American, 3 Hispanic, 2 other |  |  |
| Bradley et al., 2011^27^ | 3.85 | USA |  | no | mcrpc | 44 | 500mg cilengitide + bisphosphonates | 2000mg cilengitide + bisphosphonates |  |  | 38 white, 4 African American, 2 Asian |  |  |
| Chadha et al., 2010^28^ | 6.07 | USA |  | no | mcrpc | 18 | oral dexamethasone and calcitriol |  |  |  | 15 Caucasian |  |  |
| Chen et al., 2012^29^ | 5.59 | USA | NCT00225420 | no | T1c-T3 Gleeson 7-10 | 18 | Weekly docetaxel chemotherapy (to existing high-dose intensity modulated radiation therapy (IMRT) and androgen-deprivation therapy (ADT)) |  |  |  | 14 Caucasian 4 African American |  |  |
| Chu et al., 2015^30^ | 9.16 | USA |  | no | non metastatic prostate cancer | 127 | bicalutamide plus placebo | bicalutamide plus dutasteride |  |  | 101 white, 21 African American, 3 American Indian or Alaska native, 2 Asian |  |  |
| Corn et al., 2019^31^ | 41.3 | USA | NCT01505868 | no | mcrpc | 160 | Cabazitaxel | Cabazitaxel and carboplatin |  |  | 121 white, 24 black, 15 other |  |  |
| DiPaola et al., 2010^32^ | 4.20 | USA | CDR0000067865 | no | mcrpc | 63 | MEV | CRA/IFN/TAX |  |  | 58 white, 4 black, 1 Hispanic |  |  |
| Dorff et al., 2019^33^ | 2.45 | USA | NCT01685125 | no | mcrpc | 26 | Abiraterone | Abiraterone + Dasatinib |  |  | 11 Caucasian, 9 Hispanic, 3 black, 3 Asian |  |  |
| Dreicer et al., 2013^34^ | 3.85 | USA | NCT00466440 | no | mcrpc | 94 | DPE | DPP |  |  | 72 Caucasian, 15 African American, 6 Hispanic, 1 east Asian |  |  |
| Freedland al., 2013^35^ | 3.47 | USA | NCT00719030 | no | prostate adenocarcinoma | 69 | POMx | placebo |  |  | 58 white, 10 black, 1 native American |  |  |
| Heath et al., 2019^36^ | 5.55 | USA | NCT00527124 | no | mcrpc | 58 | DP+C | DP |  |  | 35 white, 19 black, 2 other |  |  |
| Hussain et al., 2015^37^ | 9.16 | USA | NCT00683475 | no | mcrpc | 138 | Cixutumumab + M + P | Ramucirumab+M+P |  |  | 119 white, 10 black or African American, 3 other |  |  |
| Jones et al., 2011^38^ | 91.2 | USA | NCT00002597 | no | T1b, T1c, T2a, or T2b prostate adenocarcinoma | 1979 | Short-Term ADT plus Radiotherapy | Radiotherapy Alone |  |  | 1501 white, 395 black, 53 Hispanic, 30 other or unknown |  |  |
| Koshkin et al., 2019^39^ | 3.85 | USA | NCT00526591 | no | localized high risk | 17 | high dose | low dose |  |  | 16/17 Caucasian |  |  |
| Kumar et al., 2020^40^ | 3.33 | USA | N/A | no | localized | 71 | isoflavones | placebo |  |  | 53 white, 15 black or African American, 7 Hispanic |  |  |
| Lee et al., 2016^41^ | 44.5 | USA |  | no | low risk PC | 1115 | 3D-CRT/IMRT 73.8 Gy | 3D-CRT/IMRT 70 Gy |  |  | 866 white, 6 American Indian/Alaska native, 15 Asian, 190 black or African American, 2 native Hawaiian or other pacific islander |  |  |
| Lin et al., 2013^42^ | 3.50 | USA |  | no | biochemically recurrent hormone-naïve prostate cancer | 47 | low dose | high dose |  |  | 42 white, 5 non-white |  |  |
| Monk et al., 2015^43^ | 44.5 | USA |  | no | mcrpc | 78 | Tivantinib | placebo |  |  | 69 Caucasian, 8 African American, 1 Asian |  |  |
| Nguyen et al., 2012^44^ | 3.47 | USA | NCT00459407 | no |  | 50 | polyphenon E | placebo |  |  | 45 white, 1 native American, 2 multiracial |  |  |
| Parsons et al., 2018^45^ | 18.7 | USA | NCT02326805 | no | localized | 154 | placebo | PROSTVAC |  |  | 133 white, 3 Asian, 1 native Hawaiian, 11 black or African American, 1 multiple, 5 unknown, 143 non-Hispanic, 5 Hispanic, 6 unknown |  |  |
| Rosenthal et al., 2015^46^ | 7.04 | USA | Radiation Therapy Oncology Group 9902 | no | localized (T≥2) | 397 | 2 Years of Androgen Suppression (AS) and Radiation Therapy (RT) without Adjuvant Chemotherapy | 2 Years of Androgen Suppression (AS) and Radiation Therapy (RT) with Adjuvant Chemotherapy |  |  | 276 white, 7 Hispanic, 107 African American, 1 native Hawaiian, 3 Asian, 2 American Indian or Alaska native |  |  |
| Small et al., 2015^47^ | 12.5 | USA | NCT01487863 | no | mcrpc | 69 | Sipuleucel-T with Concurrent Abiraterone Acetate plus Prednisone | Sipuleucel-T versus Sequential Abiraterone Acetate plus Prednisone |  |  | 62 Caucasian, 2 Asian, 5 black or African American |  |  |
| Stein et al., 2018^48^ | 3.50 | USA | NCT02737332 | no | mcrpc | 53 | abiraterone acetate fine particle formulation | originator abiraterone acetate |  |  | 40 white, 1 Asian, 11 black, 1 other, 6 Hispanic/Latino |  |  |
| Stein et al., 2019^49^ | 2.34 | USA |  | no | mcrpc | 116 | Eribulin mesylate |  |  |  | 108 Caucasian, 5 black, 1 Asian, 1 native American, 1 unknown |  |  |
| Wei et al., 2017^50^ | 44.5 | USA | NCT02215161 | no | mcrpc | 14 | Selinexor |  |  |  | 12 white, 1 Asian, 1 unknown |  |  |
| Wilt et al., 2020^51^ | 18.7 | USA | NCT00007645 | no | T1-T2 Nx M0 | 731 | Radical prostatectomy | observation |  |  | 452 white, 233 black, 47 other |  |  |
| Zietman et al., 2010^52^ | 44.5 | USA | NCT00002703 | no | T1b-c or T2a-b No T1a No T1b-c | 394 | Conventional dose radiation | High dose radiation |  |  | 353 white, 11 Hispanic, 17 black, 10 other |  |  |
| Fizazi et al., 2015^53^ | 44.5 | USA | [NCT01193257](http://clinicaltrials.gov/show/NCT01193257) | no | mcrpc | 1099 | Orteronel Plus Prednisone | Placebo Plus Prednisone |  |  | 925 white, 27 black/African American, 125 Asian, 17 other |  |  |
| Kumar 2010^54^ | N/A | USA | PMID: 20205984 | no | localized | 45 | isoflavone 40mg | isoflavone 60mg | isoflavone 80mg | control | 42 white, 1 black, 1 other Hispanic vs non-Hispanic also reported |  |  |
| 2019a^55^ | 44.5 | USA | NCT01683994 | yes | mcrpc | 45 | cabozantinib + docetaxel + prednisolone | docetaxel + prednisolone |  |  | 14 white, 11 black or African American, 1 unknown, Hispanic vs non-Hispanic also reported |  |  |
| Fizazi et al., 2020^56^ | 91.2 | Multinational | NCT02200614 | yes | not metastatic castration resistant (PSA doubling time < 10mths) | 1509 | Darolutamide | placebo |  |  | 1194 white, 193 Asian, 52 black, 55 missing, 15 other |  |  |
| Fizazi et al., 2015^57^ | 44.5 | USA | [NCT01193257](http://clinicaltrials.gov/show/NCT01193257) | no | mcrpc | 1099 | Orteronel Plus Prednisone | Placebo Plus Prednisone |  |  | 925 white, 27 black/African American, 125 Asian, 17 other |  |  |
| Marshall et al., 2020^58^ | 44.5 | USA | NCT02463799 | yes | M1 | 32 | Sipuleucel-T and radium 223 | Sipuleucel-T |  |  | 21 white, 8 black or African American, 2 Asian, 1 more than one race |  |  |
| Maughan et al., 2020^59^ | 44.5 | USA | NCT02199197 | yes | mcrpc | 49 | Radium Ra 223 Dichloride and Enzalutamide | Enzalutamide Alone |  |  | 48 white, 1 Asian |  |  |
| McNeel et al., 2015^60^ | 3.45 | USA |  | yes | mcrpc | 10 | Prostvac-VF followed by docetaxel @ month 3 | docetaxel chemotherapy alone |  |  | 9 white, 1 unknown |  |  |
| Morris et al., 2019b^61^ | 44.5 | USA | NCT01949337 | yes | progressive, castration resistant prostate cancer | 1311 | Enzalutamide | Enzalutamide, Abiraterone, Prednisone |  |  | 1088 white, 4 American indian or alaska native, 28 Asian, 2 native hawaiian, 162 black or African American, 27 unknown |  |  |
| Nct, 2011c,^62^ | NA | USA | NCT01322490 | N/A | mcrpc | 1297 | arm v | arm vg | arm p |  | 1207 white, 1 American indian or alaska native, 20 Asian, 1 native hawaiian, 65 black or African American, 3 unknown |  |  |
| Nct, 2013c,^63^ | NA | USA | NCT01804465 | N/A | mcrpc | 50 | Immediate IpilimumabTreatment | Delayed IpilimumabTreatment |  |  | 40 white, 3 Asian, 3 black or African American, 4 unknown |  |  |
| Nct, 2014b,^64^ | NA | Multinational - East and West | NCT02257736 | N/A | mcrpc | 982 | Apalutamide + Abiraterone Acetate - Prednisolone | Placebo+ Abiraterone Acetate - Prednisolone |  |  | 738 white, 17 American indian or alaska native, 111 Asian, 37 black, 39 other |  |  |
| Nct, 2014e,^65^ | NA | USA and Canada | NCT02083185 | N/A | Any adenocarcinoma | 136 | Relugolix 80 mg | Relugolix 120 mg | Leuprorelin 22.5 mg |  | 112 white, 19 black, 3 Asian |  |  |
| Nct, 2016b,^66^ | NA | USA | NCT02685267 | N/A | mcrpc | 9 | Docetaxel/Prednisone + Enzalutamide | Docetaxel/Prednisone |  |  | 5 white, 4 black or African American |  |  |
| Nct, 2016d,^67^ | NA | USA and Canada | NCT02799745 | N/A | T1c-T2a, PSA<10, N0, M0 | 227 | Enzalutamide | Active surveillance |  |  | 204 white, 21 black or African American, 1 Asian |  |  |
| Nct, 2016f,^68^ | NA | Multinational - East and West | NCT02987543 PROfound | N/A | mcrpc | 387 | Cohort A Olaparib 300mg bd | Cohort A Investigators Choice of NHA | Cohort B Olaparib 300mg bd | Cohort B Investigators Choice of NHA | 248 white, 8 black or African American, 105 Asian, 3 other |  |  |
| Palmbos et al., 2018^69^ | 44.5 | USA | NCT02059213 | yes | M1 | 62 | ADT + Ibrance® | ADT Alone |  |  | 55 not Hispanic or latino, 5 Hispanic or latino |  |  |
| Freytag 2014^70^ | 7.04 | USA | NCT00583492 | yes | T1/T2 intermediate risk | 44 | arm 1 | arm 2 |  |  | 31 white, 12 African American, 1 other |  |  |
| Powles et al., 2017^71^ | 44.5 | Multinational - East and West | NCT03016312 | yes | crpc | 771 | Atezolizumab + Enzalutamide | Enzalutamide |  |  | 577 white, 1 American indian or alaska native, 136 Asian, 1 native hawaiian, 15 black or African American, 2 more than one race |  |  |
| Roach et al., 2018^72^ | 41.3 | USA and Canada | NCT00769548 | no | localized (Any stage with risk of node involvement >15%) | 1322 | Neoadjuvant total androgen suppression (TAS) (NHT) given 2 months before and during radiation therapy (RT) to the whole pelvis followed by a prostate boost. Drug: flutamide Drug: goserelin acetate Radiation: low-LET photon therapy | Neoadjuvant TAS (NHT) given 2 months before and during RT to the prostate only. Drug: flutamide Drug: goserelin acetate Radiation: low-LET photon therapy | RT to the whole pelvis followed by a boost to the prostate followed by 4 months of TAS (AHT) Drug: flutamide Drug: goserelin acetate Radiation: low-LET photon therapy | RT to the prostate only followed by 4 months of TAS (AHT) Drug: flutamide Drug: goserelin acetate Radiation: low-LET photon therapy | 881 white, 37 Hispanic or latino, 322 black or African-American |  |  |
| Hussain et al., 2018^73^ | 91.2 | Multinational - East and West | NCT02003924 PROSPER | no | Adenocarcinoma of the prostate | 1401 | Enzalutamide 160 mg | Placebo |  |  | 991 white, 230 Asian, 31 black, 5 native hawaiian, 8 multiple, 20 other |  |  |
| Vaishampayan et al., 2018a^74^ | 33.0 | USA | not stated | yes | metastatic hormone sensitive PC | 71 | enzalutamide + ADT | bicalutamide + ADT |  |  | 41 Caucasian, 29 African American, 1 Asian |  |  |

^1^van Die MD, Williams SG, Emery J, Bone KM, Taylor JMG et al. A Placebo-Controlled Double-Blinded Randomized Pilot Study of Combination Phytotherapy in Biochemically Recurrent Prostate Cancer. *Prostate* 2017; **77**: 765-775.

^2^Yaxley JW, Coughlin GD, Chambers SK, Occhipinti S, Samaratunga H, Zajdlewicz L et al. Robot-assisted laparoscopic prostatectomy versus open radical retropubic prostatectomy: early outcomes from a randomised controlled phase 3 study." *Lancet* 2016; **388**: 1057-1066.

^3^Re: cabazitaxel versus Docetaxel as First-Line Therapy for Patients with Metastatic Castration-Resistant Prostate Cancer: a Randomized Phase III Trial-FIRSTANA. *J Urol* 2018; **199**: 893‐894.

^4^Matsubara NG, Kimura H, Uemura H, Uemura M, Nakamura S, Nagamori A et al. A randomized, double-blind, comparison of radium-223 and placebo, in combination with abiraterone acetate and prednisolone, in castration-resistant metastatic prostate cancer: subgroup analysis of Japanese patients in the ERA 223 study*. Int J Clin Onc* 2019;.

^5^Beer TM, Kwon ED, Drake CG, Fizazi K, Logothetis C, Gravis G, et al. Randomized, Double-Blind, Phase III Trial of Ipilimumab Versus Placebo in Asymptomatic or Minimally Symptomatic Patients with Metastatic Chemotherapy-Naive Castration-Resistant Prostate Cancer. *J Clinic Onc* 2017; **35**: 40-47.

^6^De Bono JSS, Oudard M, Ozguroglu S, Hansen JP, Machiels I, Kocak G, et al. Prednisone plus cabazitaxel or mitoxantrone for metastatic castration-resistant prostate cancer progressing after docetaxel treatment: a randomised open-label trial. *Lancet* 2010; **376**: 1147‐1154.

^7^De Bono JSS, Piulats M, Pandha HS, Petrylak DP, Saad F, Aparicio LMA, et al. Phase II randomized study of figitumumab plus docetaxel and docetaxel alone with crossover for metastatic castration-resistant prostate cancer. *Clin Cancer Res* 2014; **20**: 1925-1934.

^8^Fizazi K, Ulys A, Sengelov L, Moe M, Ladoire S, Thiery-Vuillemin A, et al. A randomized, double-blind, placebo-controlled phase II study of maintenance therapy with tasquinimod in patients with metastatic castration-resistant prostate cancer responsive to or stabilized during first-line docetaxel chemotherapy. *Ann Oncol* 2017; **28**: 2741-2746.

^9^Hakenberg OW, Perez-Gracia JL, Castellano D, Demkow T, Ali T, Caffo A, et al. Randomised phase II study of second-line olaratumab with mitoxantrone/prednisone versus mitoxantrone/prednisone alone in metastatic castration-resistant prostate cancer. *Eur J Cancer* 2019; **107**: 186‐195.

^10^Heidenreich ASK, Rawal K, Szkarlat N, Bogdanova L, Dirix A, Stenzl M, et al. A randomized, double-blind, multicenter, phase 2 study of a human monoclonal antibody to human αν integrins (intetumumab) in combination with docetaxel and prednisone for the first-line treatment of patients with metastatic castration-resistant prostate cancer. *Ann Oncol* 2013; **24**: 329‐336.

^11^Pili RM, Häggman WM, Stadler JR, Gingrich VJ, Assikis A, Björk O, et al. Phase II randomized, double-blind, placebo-controlled study of tasquinimod in men with minimally symptomatic metastatic castrate-resistant prostate cancer. *J Clin Oncol* 2011; **29**: 4022‐4028.

^12^Shore NDS, Chowdhury A, Villers L, Klotz DR, Siemens N, Phung S, et al. Efficacy and safety of enzalutamide versus bicalutamide for patients with metastatic prostate cancer (TERRAIN): a randomised, double-blind, phase 2 study. *Lancet Oncol* 2016; **17**: 153‐163.

^13^Smith MJ, De Bono C, Sternberg S, Le Moulec S, Oudard U, De Giorgi M et al. Phase III Study of Cabozantinib in Previously Treated Metastatic Castration-Resistant Prostate Cancer: COMET-1. *J Clin Oncol* 2016; **34**: 3005‐3013.

^14^Sonpavde GV, Matveev JM, Burke JR, Caton MT, Fleming TE, Hutson MD, et al. Randomized phase II trial of docetaxel plus prednisone in combination with placebo or AT-101, an oral small molecule Bcl-2 family antagonist, as first-line therapy for metastatic castration-resistant prostate cancer. *Ann Oncol* 2012; **23**: 1803-1808.

^15^Tannock IF, Fizazi K, Ivanov S, Karlsson CT, Flechon A, Skoneczna I, et al. Aflibercept versus placebo in combination with docetaxel and prednisone for treatment of men with metastatic castration-resistant prostate cancer (VENICE): a phase 3, double-blind randomised trial. *Lancet Oncol* 2013; **14**: 760-768.

^16^Wiechno PBG, Somer B, Mellado PL, Chlosta JM, Cervera Grau D, Castellano C, et al. A randomised phase 2 study combining LY2181308 sodium (survivin antisense oligonucleotide) with first-line docetaxel/prednisone in patients with castration-resistant prostate cancer. *Eur Urol* 2014; **65**: 516-520.

^17^Ye DY, Huang F, Zhou K, Xie V, Matveev C, Li B, et al. A phase 3, double-blind, randomized placebo-controlled efficacy and safety study of abiraterone acetate in chemotherapy-naive patients with mCRPC in China, Malaysia, Thailand and Russia. *Asian Journal of Urology* 2017; **4**: 75-85.

^18^Yu EY, Li H, Higano CS, Agarwal N, Pal SK, Alva A, et al. SWOG S0925: A Randomized Phase II Study of Androgen Deprivation Combined with Cixutumumab Versus Androgen Deprivation Alone in Patients With New Metastatic Hormone-Sensitive Prostate Cancer. *J Clin Oncol* 2015; **33**: 1601-1608.

^19^Yu EY, Ellard SL, Hotte SJ, Gingerich JR, Joshua AM, Gleave MS et al. A randomized phase 2 study of a HSP27 targeting antisense, apatorsen with prednisone versus prednisone alone, in patients with metastatic castration resistant prostate cancer. *Invest New Drugs* 2018; **36**: 278‐287.

^20^Hussain M, Fizazi K, Saad F, Rathenborg P, Shore N, Ferreira U, et al. Enzalutamide in Men with Nonmetastatic, Castration-Resistant Prostate Cancer. *N Engl J Med* 2018; **378**:2465-74.

^21^Roach M, Moughan J, Lawton CAF, Dicker AP, Zeitzer KL, Gore EM, et al. Sequence of hormonal therapy and radiotherapy field size in unfavourable, localised prostate cancer (NRG/RTOG 9413): long-term results of a randomised, phase 3 trial. *Lancet Oncol* 2018; **19**:1504-15.

^22^Sternberg 2016 – Randomized, double-blind, placebo-controlled phase III study of tasquinimod in men with metastatic castration-resistant prostate cancer. J Clin Oncol 2016; **34**:2636-2643.

^23^Clarke NP, Wiechno B, Alekseev N, Sala R, Jones I, Kocak VE, et al. Olaparib combined with abiraterone in patients with metastatic castration-resistant prostate cancer: a randomised, double-blind, placebo-controlled, phase 2 trial. *Lancet Oncol* 2018; **19**: 975‐986.

^24^Antonarakis ES, Tagawa ST, Galletti G, Worroll D, Ballman K, Vanhuyse M, et al. Randomized, Noncomparative, Phase II Trial of Early Switch from Docetaxel to Cabazitaxel or Vice Versa, With Integrated Biomarker Analysis, in Men With Chemotherapy-Naïve, Metastatic, Castration-Resistant Prostate Cancer. *J Clin Oncol* 2017; **35**: 3181‐3188.

^25^Beard CWB, Stason Q, Wang J, Manola E, Dean-Clower JA, Dusek S, et al. Effects of complementary therapies on clinical outcomes in patients being treated with radiation therapy for prostate cancer. *Cancer* 2011; **117**: 96‐102.

^26^Beer TM, Schellhammer PF, Corman JM, Glodé LM, Hall SJ, Whitmore JB, et al. Quality of life after sipuleucel-T therapy: results from a randomized, double-blind study in patients with androgen-dependent prostate cancer. *Urology* 2013; **82**: 410‐415.

^27^Bradley DA, Daignault S, Ryan CJ. Cilengitide (EMD 121974, NSC 707544) in asymptomatic metastatic castration resistant prostate cancer patients: a randomized phase II trial by the prostate cancer clinical trials consortium. *Investigational New Drugs* 2011; **29**: 1432–40

^28^Chadha MK, Tian L, Mashtare T. Phase 2 trial of weekly intravenous 1,25 dihydroxy cholecalciferol (Calcitriol) in combination with dexamethasone for castration-resistant prostate cancer. *Cancer* 2010; **116**: 2132-2139.

^29^Chen RC, Rosenman JG, Hoffman LG. Phase I study of concurrent weekly docetaxel, high-dose intensity-modulated radiation therapy (IMRT) and androgen-deprivation therapy (ADT) for high-risk prostate cancer. *BJU International* 2012; **110**: E721–6.

^30^Chu FM, Sartor O, Gomella L, Rudo T, Somerville MC, Hereghty B et al. A randomised, double-blind study comparing the addition of bicalutamide with or without dutasteride to GnRH analogue therapy in men with non-metastatic castrate-resistant prostate cancer. *Eur J Cancer* 2015; **51**:1555-69.

^31^Corn PG, Heath EI, Zurita A, Ramesh N, Xiao L, Sei E, et al. Cabazitaxel plus carboplatin for the treatment of men with metastatic castration-resistant prostate cancers: a randomised, open-label, phase 1-2 trial. *Lancet Oncol* 2019; **20**: 1432-1443.

^32^Dipaola RS, Chen Y-H, Stein M. A randomized phase II trial of mitoxantrone, estramustine and vinorelbine or bcl-2 modulation with 13-cis retinoic acid, interferon and paclitaxel in patients with metastatic castrate-resistant prostate cancer: ECOG 3899. *Journal of Translational Medicine* 2010; **8**: 20.

^33^Dorff TB, Quinn DI, Pinski JK, Goldkorn A, Sadeghi S, Tsao-Wei D, et al. Randomized Phase II Trial of Abiraterone Alone or With Dasatinib in Men With Metastatic Castration-resistant Prostate Cancer (mCRPC). *Clin Genitourin Cancer* 2019; **17**:241-247.

^34^Dreicer R, Garcia J, Rini B, Vogelzang N, Srinivas S, Somer B, et al. A randomized, double-blind, placebo-controlled, Phase II study with and without enzastaurin in combination with docetaxel-based chemotherapy in patients with castration-resistant metastatic prostate cancer. *Invest New Drugs* 2013; **31**:1044-50.

^35^Freedland SJ, Carducci M, Kroeger N. A Double-Blind, Randomized, Neoadjuvant Study of the Tissue Effects of POMx Pills in Men with Prostate Cancer Before Radical Prostatectomy. *Cancer Prevention Research* 2013; **6**: 1120–7.

^36^Heath E, Heilbrun L, Mannuel H. Phase II, Multicenter, Randomized Trial of Docetaxel plus Prednisone with or Without Cediranib in Men with Chemotherapy-Naive Metastatic Castrate-Resistant Prostate Cancer. *The Oncologist* 2019; **24**: 1149–e807.

^37^Hussain M, Rathkopf D, Liu G. A randomised non-comparative phase II trial of cixutumumab (IMC-A12) or ramucirumab (IMC-1121B) plus mitoxantrone and prednisone in men with metastatic docetaxel-pretreated castration-resistant prostate cancer. *European Journal of Cancer* 2015; **51**: 1714–24.

^38^Jones CU, Hunt D, Mcgowan DG. Radiotherapy and Short-Term Androgen Deprivation for Localized Prostate Cancer. *New England Journal of Medicine* 2011; **365**: 107–18.

^39^Koshkin VS, Mir MC, Barata P. Randomized phase II trial of neoadjuvant everolimus in patients with high-risk localized prostate cancer. *Investigational New Drugs* 2019; **37**: 559–66.

^40^Kumar NB, Pow-Sang J, Spiess P, Dickinson S, Schell MJ. A phase II randomized clinical trial using aglycone isoflavones to treat patients with localized prostate cancer in the pre-surgical period prior to radical prostatectomy. *Oncotarget* 2020; **11**: 1218–34.

^41^Lee WR, Dignam JJ, Amin MB. Randomized Phase III Noninferiority Study Comparing Two Radiotherapy Fractionation Schedules in Patients With Low-Risk Prostate Cancer. *Journal of Clinical Oncology* 2016; **34**: 2325–32.

^42^Lin J, Zahurak M, Beer TM. A non-comparative randomized phase II study of 2 doses of ATN-224, a copper/zinc superoxide dismutase inhibitor, in patients with biochemically recurrent hormone-naïve prostate cancer. *Urologic Oncology: Seminars and Original Investigations* 2013; **31**: 581–8.

^43^Monk P, Liu G, Stadler WM. Phase II randomized, double-blind, placebo-controlled study of tivantinib in men with asymptomatic or minimally symptomatic metastatic castration-resistant prostate cancer (mCRPC). *Investigational New Drugs* 2018; **36**: 919–26.

^44^Nguyen MM, Ahmann FR, Nagle RB. Randomized, Double-Blind, Placebo-Controlled Trial of Polyphenon E in Prostate Cancer Patients before Prostatectomy: Evaluation of Potential Chemopreventive Activities. *Cancer Prevention Research* 2012; **5**: 290–8.

^45^Parsons JK, Pinto PA, Pavlovich CP, Uchio E, Kim HL, Nguyen MN, et al. A Randomized, Double-blind, Phase II Trial of PSA-TRICOM (PROSTVAC) in Patients with Localized Prostate Cancer: The Immunotherapy to Prevent Progression on Active Surveillance Study. *Eur Urol Focus* 2018; **4**:636-638.

^46^Rosenthal SA, Hunt D, Sartor AO. A Phase 3 Trial of 2 Years of Androgen Suppression and Radiation Therapy With or Without Adjuvant Chemotherapy for High-Risk Prostate Cancer: Final Results of Radiation Therapy Oncology Group Phase 3 Randomized Trial NRG Oncology RTOG 9902. *Int J Radiat Oncol Biol Phys* 2015; **93**: 294–302.

^47^Small EJ, Lance RS, Gardner TA, Karsh LI, Fong L, McCoy C, et al. A Randomized Phase II Trial of Sipuleucel-T with Concurrent versus Sequential Abiraterone Acetate plus Prednisone in Metastatic Castration-Resistant Prostate Cancer. *Clin Cancer Res* 2015; **21**: 3862-9.

^48^Stein CA, Levin R, Given R, Higano CS, Nemeth P, Bosch B, et al. Randomized phase 2 therapeutic equivalence study of abiraterone acetate fine particle formulation vs. originator abiraterone acetate in patients with metastatic castration-resistant prostate cancer: The STAAR study. *Urol Oncol* 2018; **36**: 81.e9-81.e16.

^49^Stein MN, Chen YH, Carducci MA, Hudes GR, Lerma PM, Tan WW, et al. Phase II Trial of Eribulin in Patients With Metastatic Hormone Refractory Prostate Cancer: A Trial of the ECOG-ACRIN Cancer Research Group (E5805). *Am J Clin Oncol* 2019; **42**:375-381.

^50^Wei XX, Siegel AP, Aggarwal R. A Phase II Trial of Selinexor, an Oral Selective Inhibitor of Nuclear Export Compound, in Abiraterone- and/or Enzalutamide-Refractory Metastatic Castration-Resistant Prostate Cancer. *The Oncologist* 2018; **23**: 656–e64.

^51^Wilt TJ, Vo TN, Langsetmo L, Dahm P, Wheeler T, Aronson WJ, et al. Radical Prostatectomy or Observation for Clinically Localized Prostate Cancer: Extended Follow-up of the Prostate Cancer Intervention Versus Observation Trial (PIVOT). *Eur Urol* 2020; **77**:713-724.

^52^Zietman AL, Bae K, Slater JD. Randomized Trial Comparing Conventional-Dose With High-Dose Conformal Radiation Therapy in Early-Stage Adenocarcinoma of the Prostate: Long-Term Results From Proton Radiation Oncology Group/American College of Radiology 95-09. *Journal of Clinical Oncology* 2010; **28**: 1106–11.

^53^Fizazi K, Jones R, Oudard S. Phase III, Randomized, Double-Blind, Multicenter Trial Comparing Orteronel (TAK-700) Plus Prednisone With Placebo Plus Prednisone in Patients With Metastatic Castration-Resistant Prostate Cancer That Has Progressed During or After Docetaxel-Based Therapy:. *Journal of Clinical Oncology* 2015; **33**: 723–31.

^54^Kumar NB, Kang L, Pow-Sang J, Xu P, Allen K, Riccardi D, et al. Results of a randomized phase I dose-finding trial of several doses of isoflavones in men with localized prostate cancer: administration prior to radical prostatectomy. *J Soc Integr Oncol* 2010; **8**:3-13.

^55^Euctr. a multinational, randomised, double-blind, placebo-controlled, phase iii efficacy and safety study of odm-201 in men with high-risk non-metastatic castration-resistant prostate cancer. 2015.

^56^Fizazi K, Shore N, Tammela TL. Darolutamide in Nonmetastatic, Castration-Resistant Prostate Cancer. *New England Journal of Medicine* 2019; **380**: 1235–46.

^57^Fizazi K, Jones R, Oudard S. Phase III, Randomized, Double-Blind, Multicenter Trial Comparing Orteronel (TAK-700) Plus Prednisone With Placebo Plus Prednisone in Patients With Metastatic Castration-Resistant Prostate Cancer That Has Progressed During or After Docetaxel-Based Therapy. *Journal of Clinical Oncology* 2015; **33**: 723–31.

^58^Marshall CH, Park JC, DeWeese TL, King S, Afful M, Hurrelbrink J, et al. Randomized phase II study of sipuleucel-T (SipT) with or without radium-223 (Ra223) in men with asymptomatic bone-metastatic castrate-resistant prostate cancer (mCRPC). *J Clin Oncol* 2020; Conference **38**.

^59^Maughan BL, Hahn AW, Nussenzveig R, Hoffman J, Morton K, Gupta S, et al. Randomized phase II trial of radium-223 (RA) plus enzalutamide (EZ) versus EZ alone in metastatic castration-refractory prostate cancer (mCRPC): Long-term follow up of secondary endpoints. *J Clin Oncol* 2020; Conference **38**.

^60^McNeel DG, Chen YH, Gulley JL, Dwyer AJ, Madan RA, Carducci MA, et al. Randomized phase II trial of docetaxel with or without PSA-TRICOM vaccine in patients with castrate-resistant metastatic prostate cancer: a trial of the ECOG-ACRIN cancer research group (E1809). *Hum Vaccin Immunother* 2015; **11**: 2469‐2474.

^61^Morris MJ, Heller G, Bryce AH, Armstrong AJ, Beltran H, Hahn OM, et al. Alliance A031201: A phase III trial of enzalutamide (ENZ) versus enzalutamide, abiraterone, and prednisone (ENZ/AAP) for metastatic castration resistant prostate cancer (mCRPC). *J Clin Oncol* 2019. Conference **37**.

^62^Nct 2011. A Randomized, Double-blind, Phase 3 Efficacy Trial of PROSTVAC-V/F +/- GM-CSF in Men with Asymptomatic or Minimally Symptomatic Metastatic Castrate-Resistant Prostate Cancer. https://clinicaltrials.gov/ct2/show/NCT01322490

^63^Nct 2013. A Randomized Phase 2 Trial of Combining Sipuleucel-T With Immediate vs. Delayed CTLA-4 Blockade for Prostate Cancer. https://clinicaltrials.gov/show/NCT01804465.

^64^Nct 2014. An Efficacy and Safety Study of Apalutamide (JNJ-56021927) in Combination With Abiraterone Acetate and Prednisone Versus Abiraterone Acetate and Prednisone in Participants With Chemotherapy-naive Metastatic Castration-resistant Prostate Cancer (mCRPC). https://clinicaltrials.gov/show/NCT02257736.

^65^Nct 2014. A Phase 2 Study to Evaluate the Safety and Efficacy of TAK-385, Together with a Leuprorelin Observational Cohort, in Participants With Prostate Cancer. https://clinicaltrials.gov/show/NCT02083185.

^66^Nct 2016. Docetaxel/Prednisone Versus Docetaxel/Prednisone and Enzalutamide in Castration-Resistant Prostate Cancer. https://clinicaltrials.gov/show/NCT02685267.

^67^Nct 2016. A Randomized Study of Enzalutamide in Patients with Localized Prostate Cancer Undergoing Active Surveillance. https://clinicaltrials.gov/show/NCT02799745.

^68^Nct 2016. Study of Olaparib (Lynparza™) Versus Enzalutamide or Abiraterone Acetate in Men with Metastatic Castration-Resistant Prostate Cancer (PROfound Study). https://clinicaltrials.gov/show/NCT02987543.

^69^Palmbos PL, Tomlins SA, Agarwal N, Twardowski P, Morgans AK, Kelly WK, et al. Cotargeting AR signaling and cell cycle: a randomized phase II study of androgen deprivation therapy with or without palbociclib in RB-positive metastatic hormone sensitive prostate cancer (mHSPC). *J Clin Oncol* 2018; **36**.

^70^Freytag SO, Stricker H, Lu M, Elshaikh M, Aref I, Pradhan D, et al. Prospective randomized phase 2 trial of intensity modulated radiation therapy with or without oncolytic adenovirus-mediated cytotoxic gene therapy in intermediate-risk prostate cancer. *Int J Radiat Oncol Biol Phys* 2014; **89**:268-76.

^71^Powles TK, Fizazi S, Gillessen CG, Drake DE, Rathkopf S, Narayanan MC, et al. A phase III trial comparing atezolizumab with enzalutamide vs enzalutamide alone in patients with metastatic castration-resistant prostate cancer (mCRPC). *J Clin Oncol* 2017; **35**.

^72^Roach M, Moughan J, Lawton CAF, Dicker AP, Zeitzer KL, Gore EM, et al. Sequence of hormonal therapy and radiotherapy field size in unfavourable, localised prostate cancer (NRG/RTOG 9413): long-term results of a randomised, phase 3 trial. *Lancet Oncol* 2018; **19**:1504-15.

^73^Hussain M, Fizazi K, Saad F, Rathenborg P, Shore N, Ferreira U, et al. Enzalutamide in Men with Nonmetastatic, Castration-Resistant Prostate Cancer. *N Engl J Med* 2018; **378**:2465-74.

^74^Vaishampayan UN, Heilbrun L, Monk P, Sonpavde G, Tejwani S, Heath EI, et al. Randomized trial of androgen deprivation therapy (ADT) + enzalutamide (Arm A) versus ADT + bicalutamide (Arm B) in metastatic hormone sensitive prostate cancer (mHSPC). Ann Oncol 2018; **29**: viii276.
